# Involvement of Calcium and Calmodulin in Nitric Oxide-Regulated Senescence of Cut Lily Flowers

**DOI:** 10.3389/fpls.2018.01284

**Published:** 2018-09-03

**Authors:** Jing Zhang, Hua Fang, Jianqiang Huo, Dengjing Huang, Bo Wang, Weibiao Liao

**Affiliations:** College of Horticulture, Gansu Agricultural University, Lanzhou, China

**Keywords:** postharvest freshness, calcium ion/calmodulin, Ca^2+^-ATPase, gene expression, calcium signaling

## Abstract

Both nitric oxide (NO) and calcium ion (Ca^2+^)/calmodulin (CaM) have been shown to regulate the senescence of cut flowers. However, not much is known about the crosstalk between NO and Ca^2+^/CaM during the senescence of cut flowers. In this study, cut Oriental × Trumpet hybrid lily “Manissa” were used to investigate the roles and relationship between NO and Ca^2+^/CaM during postharvest freshness. The results show that the effects of CaCl_2_ or NO donor SNAP on the vase life, maximum flower diameter and hours until full opening were dose-dependent, with an optimum concentration of 20 mM CaCl_2_ or 100 μM SNAP. However, Ca^2+^ chelators EGTA or BAPTA/AM, Ca^2+^ channel inhibitors LaCl_3_ or nifedipine and CaM antagonists W-7 or TFP inhibited the promotion of SNAP. SNAP applied alone significantly increased the endogenous Ca^2+^/CaM contents in cut lily flowers, while EGTA, BAPTA/AM, LaCl_3_, nifedipine, W-7, and TFP decreased the advancement of SNAP. In addition, the SNAP-induced Ca^2+^-ATPase activity was more than twice as much as the control, but EGTA, BAPTA/AM, LaCl_3_, nifedipine, W-7, and TFP also reversed the enhancement. Moreover, EGTA, BAPTA/AM, LaCl_3_, nifedipine, W-7, and TFP prevented the SNAP-induced upregulation of gene expression of *CaM*, *CBL1*, and *CBL3*, which is associated with calcium signaling pathway. Overall, these results suggest that Ca^2+^/CaM may function as downstream molecules in NO-regulated senescence of cut flowers.

## Introduction

Cut lilies (*Lilium* spp.), a bulbous plant with large trumpet-shaped and typically fragrant flowers (Liao et al., 2013), are in demand worldwide because of their superior commercial and ornamental value. However, the postharvest life of cut lilies is usually short because of wilting, color changing, abscission, and early leaf yellowing (de Almeida et al., 2017). Senescence is the main reason for the short vase life and poor quality of cut flowers, which involves a general degradation of nucleic acids, proteins, and cell membranes, as well as increased activities of RNase and other hydrolytic enzymes (Shabanian et al., 2018). These structural, biochemical, and molecular changes are also the hallmarks of programmed cell death (PCD). Therefore, ethylene-induced PCD is a critical factor of senescence for ethylene-sensitive flowers (Zhou et al., 2005). Moreover, postharvest life and quality of cut flowers are controlled by a combination of factors including multiple genetic factors, pre-harvest environmental conditions throughout the supply chain, plant maturity and season of planting and harvesting, plant nutritional status, sensitivity to ethylene and oxidative stress, and postharvest temperature fluctuations and water balance (Liao et al., 2013). Therefore, to enhance vase life and maintain quality of cut flowers, convenient, ecological, and economical approaches to decelerate senescence are needed. Furthermore, understanding the mechanism of these fresh preservation methods is of vital importance for exploring new approaches for postharvest freshness.

Previous studies have shown that nitric oxide (NO) may function as an influential plant growth regulator (Asgher et al., 2017). It is evident that NO as a signaling molecule mediates many specific developmental processes, including seed dormancy or germination, de-etiolation, hypocotyl elongation, stomatal movement, pollen tubes growth, flowering, cell wall lignification, xylem differentiation, cellulose biosynthesis, chlorophyll biosynthesis/photosynthesis, gravitropism, cell polarity, maturation, senescence, and root organogenesis (Luis et al., 2015). NO also mediates various plant abiotic responses, such as salinity, water stress, extreme heat and cold, mechanical injury, UV radiation, ozone, heavy metal toxicity, herbicide, nutrient deficiency, and among other risks (Fancy et al., 2016). A recent study found that NO increased water uptake and promoted antioxidant activity and consequently enhanced vase life of cut gerbera flowers (Shabanian et al., 2018). NO in plants is produced by a variety of enzymatic and non-enzymatic mechanisms (Benavides et al., 2016). The enzymatic biosynthesis including NO synthesis (NOS)-like enzymes, nitrite reductase (NR), xanthine oxidase/dehy-drogenase (XDH) and nitric: NO oxidoreductase (Ni-NOR) (Liao et al., 2012a). The arginine and nitrite pathways are most plausible routes in NO generation. The NOS activity has been documented in many plant species, but no cloned NOS enzyme has been identified. NR is the best described enzymatic source of NO in plants which catalyzes nitrite to NO rely on NAD(P)H (Chamizo-Ampudia et al., 2017).

As an essential cytoplasmic second messenger, calcium ion (Ca^2+^) plays critical roles in plant response to biotic and abiotic stresses, including light, unfavorable temperature, salt and osmotic stress, phytohormones, oxidative stress, wind stimulation, wounding, and anoxia. Ca^2+^ also plays an important role in plant membrane stability, cell wall stabilization, and cell integrity (Ranty et al., 2016). Moreover, Ca^2+^ was reported to delay senescence of cut rose flowers by protecting both membrane phospholipids and membrane proteins from degradation, and reducing ethylene production (Torre et al., 1999). In response to various environmental changes, Ca^2+^ signals must be decoded by several Ca^2+^ sensors or Ca^2+^ binding proteins. Calmodulins (CaMs), calcineurin B-like (CBL) proteins, and calcium-dependent protein kinase (CDPK) are three main families of Ca^2+^ sensors (Boudsocq and Sheen, 2013). CaM, one of the most ubiquitous calcium-modulated proteins, is highly conserved during evolution. CaM transmits Ca^2+^ signal through interacting target proteins and regulating their activities, and subsequently regulates many critical processes such as immunity, pollen tube growth, cell wall regeneration, cell division, defense against necrotrophic pathogens, and high temperature tolerance (Liao et al., 2013). CDPKs are versatile and evolutionarily conserved Ca^2+^ sensors/transducers that function in a diverse array of plant process in immune and stress signaling networks (Boudsocq and Sheen, 2013). CBLs also play vital roles in plant responses to diverse abiotic stress (Lu et al., 2017).

Furthermore, there is a crosstalk among NO, Ca^2+^, and CaM in plant growth and development as well as in response to abiotic stresses (Takata et al., 2011). On the one hand, the synthesis of NO by NOS is strictly Ca^2+^/CaM dependent. Ca^2+^ can act as both a promoter and a sensor of NO signaling pathways (Jeandroz et al., 2013). Similarly, Ca^2+^ signaling can induce endogenous NO accumulation during stomatal closure in *Arabidopsis* guard cells (Wang et al., 2012). On the other hand, NO regulates the gating/conductance of Ca^2+^ channels and impact the activity of Ca^2+^ pumps as well as Ca^2+^ sensors. Nearly all types of Ca^2+^ channels appear to be mediated by NO (Clementi and Meldolesi, 1997). The study on marigold indicate that exogenous NO promoted adventitious rooting by increasing endogenous Ca^2+^ and CaM levels (Liao et al., 2012b). In addition, the involvement of Ca^2+^/CaM in NO-induced adventitious rooting in cucumber under simulated osmotic stress has also been studied (Niu et al., 2017). Thus, Ca^2+^ appears to be both upstream and downstream of NO signaling during plant growth and stress responses. Therefore, there is a complex interplay that exists between Ca^2+^/CaM and NO.

It is clear that both exogenous NO and Ca^2+^ can enhance vase life and keep quality during postharvest freshness of cut flowers by inhibiting ethylene production and delaying bending, respectively (Liao et al., 2013; Perik et al., 2014). However, not much is known about the crosstalk among NO and Ca^2+^/CaM on flowers freshness. In this study, cut Oriental × Trumpet hybrid lilium “Manissa” were used to investigate the mechanism by which Ca^2+^/CaM was involved in NO-enhanced postharvest freshness.

## Materials and Methods

### Plant Materials and Treatments

Cut Oriental × Trumpet hybrid lilium “Manissa” were purchased from a wholesale cut flowers market in Lanzhou, China. Upon arrival in the laboratory, green bud stage (commercial harvest stage) of lilies were rehydrated for 2 h, then the flowering stems were recut to a uniform length of 45 cm under distilled water to avoid air embolism. The blades of cutters were surface sterilized by rinsing in 95% (v/v) ethanol prior to use. The flowers were selected for uniformity of size, color, and free from any defects. All experiments were performed in the laboratory at temperature of 20 ± 2°C, relative humidity of 60 ± 10%, and light intensity of 12 μmol m^-2^ s^-1^ from cool white florescent tubes with a daily light period of 12 h.

Experiment 1: after selecting the flowers, every 5 flowers were randomly put in a 2500 mL glass vase containing 1000 mL distilled water (control 1), 40 mM potassium chloride (KCl, control 2) or various concentrations of 1000 mL of test solutions. Unless otherwise specified, the word “control” refers to control 1 in the full text. Glass vases were surface sterilized with ethanol which was used for cutters as described above. There were two kinds of test solutions which were respectively made by NO donor S-nitro-N-acetyl-penicillamine (SNAP) or calcium dichloride (CaCl_2_). The doses of SNAP solution were 50, 100, 200, and 400 μM while the concentration grads of CaCl_2_ solution were 5, 10, 20, 40, and 60 mM. After three replications of each treatment, 100 μM SNAP or 20 mM CaCl_2_ treatment was used in the following study.

Experiment 2: 10 treatments were applied: (1) Control (distilled water); (2) 100 μM S-nitro-N-acetyl-penicillamine (SNAP); (3) 20 mM calcium dichloride (CaCl_2_); (4) 120 μM [Ethylene glycol-bis (-aminoethy ether) N, N, N′, N′-tetraacetic acid] (EGTA) + SNAP; (5) 30 μM [1, 2-bis (2-aminophenoxy) ethane–N, N, N′, N′-tetraacetic acid tetrakis (acetoxymethyl ester)] (BAPTA/AM, Tokyo Chemical Industry, Tokyo, Japan) + SNAP; (6) 500 μM lanthanum chloride (LaCl_3_, Solarbio, Beijing, China) + SNAP; (7) 150 μM nifedipine (Tokyo Chemical Industry, Tokyo, Japan) +SNAP; (8) 80 μM [N-(6-aminohexyl)-5-chloro-1-naphtalene sulfonamide] (W-7, Tokyo Chemical Industry, Tokyo, Japan)+ SNAP; (9) 100 μM trifluoperazine (TFP, Solarbio, Beijing, China) + SNAP; (10) 150 μM nifedipine. Each treatment was conducted in three replications. The concentrations of these chemicals were selected based on the result of a recent experiment conducted in our laboratory (Niu et al., 2017). Fresh test solutions were made every day to replace the vase solutions which were made the day before.

### Vase Life and Maximum Flower Diameter Determination

The vase life of each cut lily flower was determined by the number of days from the time when the flower was placed in the vase solution until flower had no decorative value (wilting, discoloration, or stem bending). The flower diameter was defined as the maximum width of each flower and measured by a vernier caliper. The hours until full flower opening (HFO) was determined by the number of hours from the time when the flower was placed into the vase solution until the flower diameter reached the maximum value. There was an observation every 6 h at the same time of a day.

### Determination of Calcium (Ca^2+^), Calmodulin (CaM), and Ca^2+^-ATPase Activity

The Ca^2+^ level in the cut lily leaves were measured by atomic absorption spectral analysis of nitric–perchloric acid digests. Soluble protein content in the extracts for CaM assay was determined according to Bradford (1976) using BSA as standard. The CaM contents were determined by its capacity to activate bovine brain cAMP phosphodiesterase as described by Ye et al. (1990) using maize germ CaM as a standard (Gong et al., 1996). The determination of Ca^2+^-ATPase activity was performed using the modified one-step lead method of Jian et al. (1999).

### Fluorescence Quantitative Real-Time PCR

Total RNA was abstracted from healthy lily leaves with TaKaRa MiniBEST plant RNA extraction kit (Takara Bio Inc., Kusatsu, Shiga, Japan) according to the manufacturer’s instructions. The concentration of total RNA was measured using an ultraviolet spectrophotometer, and the quality of RNA was determined based on its OD_260_/OD_280_ ratio. About 2 μL of DNA-free total RNA was used for synthesis cDNA with a PrimeScript RT Master Mix kit (Takara Bio Inc., Kusatsu, Shiga, Japan). Quantitative real-time PCR reactions were performed with a Mastercycler^®^ep realplex real-time PCR system (Eppendorf, Hamburg, Germany) with SYBR^®^ Premix Ex Taq^TM^ (Takara Bio Inc., Kusatsu, Shiga, Japan). The cDNA was amplified using specific primers (**Supplementary Table S1**). The amplification conditions were as follows: 95°C pre-degeneration for 30 s and repeat once, followed by 40 cycles of 95°C for 15 s, and then annealing at 60°C for 30 s. The expression levels of corresponding genes were presented as values relative to the corresponding control samples under the indicated conditions, with normalization of data to the geometric average of internal lily *actin*. Each sample was set three biological replicates.

### Statistical Analysis of the Data

All of data presented in the tables and figures were expressed at the mean value ± SE from three independent duplications (five flowering stems per replication). Data collected were subjected to analysis of variance (ANOVA), and statistical divergence among treatments was analyzed through Duncan’s multiple range test (*P* < 0.05). All statistical analysis was carried out using the statistical package for social science for windows (version13.00; SPSS, Inc., Chicago, IL, United States).

## Results

### Effects of Various Concentrations of CaCl_2_ and SNAP on Senescence

There was no difference in the vase life of cut lilies between treatments with distilled water (control 1) and KCl (control 2; **Table 1**). CaCl_2_ treatments at the concentration of 10, 20, and 40 mM significantly extended the vase life of cut lily flowers compared to both control 1 and control 2. However, when tested at 5 mM and 60 mM CaCl_2_, each of the concentration had no significant effects on vase life. The longest vase life obtained by 20 mM CaCl_2_ treatment, which was 21 and 19% longer than the control 1 and control 2, respectively. As shown in **Table 1**, exogenous CaCl_2_ at proper levels (10, 20, and 40 mM) increased the maximum flower diameter of cut lily significantly in comparison to the control. Treatment with 20 mM CaCl_2_ created the maximum value. Placing cut lily flowers in the 20 mM CaCl_2_ solution created the maximum HFO, while 5 and 60 mM did not influence HFO (**Table 1**).

**Table 1:** Effects of various concentrations of CaCl_2_ on vase life, maximum flower diameter, and hours until full flower opening of cut OT hybrids lily “Manissa.”

Treatments	Vase life (d)	Maximum flower diameter (mm)	Hours until full flower opening (HFO, h)
Distilled water (control 1)	10.2 ± 0.5 c	186.75 ± 1.17c	152.2 ± 6.9 c
40 mM KCl (control 2)	10.3 ± 0.3 c	208.30 ± 4.10b	163.9 ± 4.6 b
5 mM CaCl_2_	10.1 ± 0.2 c	201.04 ± 19.01 b	147.8 ± 14.4 c
10 mM CaCl_2_	11.3 ± 0.2 b	216.65 ± 6.05 b	167.0 ± 9.4 b
20 mM CaCl_2_	12.3 ± 0.4 a	232.12 ± 11.33 a	186.5 ± 6.8 a
40 mM CaCl_2_	11.7 ± 0.7 b	210.69 ± 5.55 b	163.8 ± 8.9 b
60 mM CaCl_2_	10.8 ± 0.4 c	186.57 ± 7.34 c	148.3 ± 2.2 c

*The values (mean ± SE) are the average of three independent experiments (n = 15 flowering stems). Values not sharing the same letters in the same list are statistically significant by Duncan’s multiple range test at 5% level.*

As **Table 2** shows, the effects of NO donor SNAP on the senescence of cut lily were dose-dependent. The vase life was obviously prolonged by lower dosage of SNAP (50, 100, and 200 μM) while higher dosage of SNAP (400 μM) was phytotoxic, thereby resulting in a shortened vase life. Treatment with 100 μM SNAP produced the longest vase life, which was about 132.29% of the control. Compared to the control, all various concentrations of treatments with SNAP markedly increased the maximum diameter (**Table 2**). Both treatments with 100 and 200 μM SNAP significantly extended the maximum flower diameter, whereas 100 μM is the best. Treatment with 100 μM SNAP extended HFO, which increased by 44.98% compared with the control. Therefore, 20 mM CaCl_2_ and 100 μM SNAP might delay senescence by slowing down flower opening, and they were used in further study in this experiment.

**Table 2:** Effects of various concentrations of a NO donor SNAP on vase life, maximum flower diameter, and hours until full flower opening of cut OT hybrids lily “Manissa.”

SNAP concentration (μM)	Vase life (d)	Maximum flower diameter (mm)	Hours until full flower opening (HFO, h)
0 (control)	9.6 ± 0.8 c	192.70 ± 11.6 c	148.3 ± 7.0 c
50	11 ± 0.6 b	206.05 ± 9.6 bc	169.4 ± 1.4 bc
100	12.7 ± 0.2 a	228.39 ± 4.1 a	215.0 ± 2.4 a
200	11.8 ± 0.4 b	219.05 ± 8.7 b	205.7 ± 6.0 ab
400	9.1 ± 0. 3 c	208.43 ± 5.5 bc	163.0 ± 10.8 b

*The values (mean ± SE) are the average of three independent experiments (n = 15 flowering stems). Values not sharing the same letters in the same list are statistically significant by Duncan’s multiple range test at 5% level.*

### Effects of Ca^2+^ Chelators, Ca^2+^ Channel Inhibitors, and CaM Antagonists on SNAP-Delayed Senescence

**FIGURE 1:**
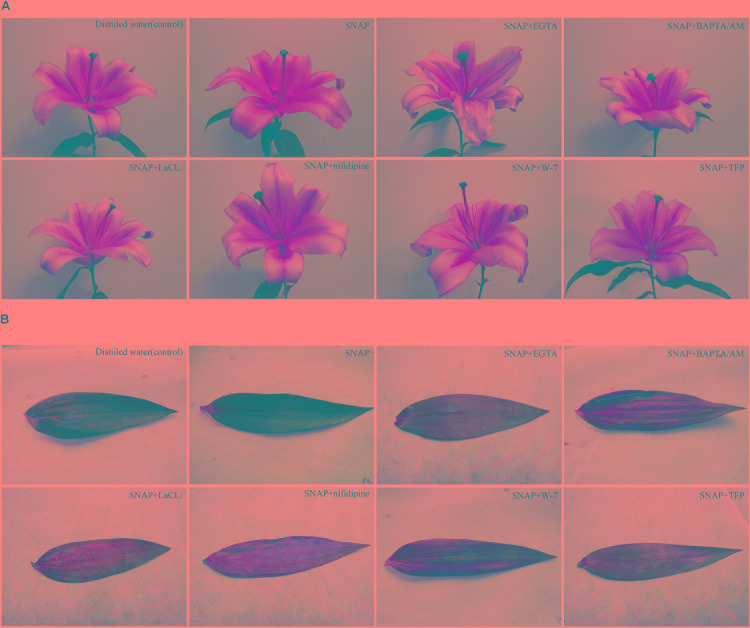
Effects of different vase solutions on lilies. Photographs of flowers **(A)** and leaves **(B)** were taken after 10 days treatments.

To further analyze whether Ca^2+^ and CaM is involved in the NO-enhanced postharvest freshness of cut lily flowers, effects of Ca^2+^ chelators (EGTA and BAPTA/AM), Ca^2+^ channel inhibitors (LaCl_3_ and nifedipine), and CaM antagonists (W-7 and TFP) co-treated with SNAP were studied (**Figure 1** and **Table 3**). When treated with SNAP solution alone, the vase life was the longest, which was 26% longer than that of the control. Treatments with SNAP plus Ca^2+^ chelators or Ca^2+^ channel inhibitors cut down the advancement of vase life, flower diameter and HFO caused by SNAP to some extent, thus produced significant effects on anti-aging of cut lily flowers. However, when CaM antagonists W-7 or TFP was added to SNAP containing solution, it reversed the SNAP-induced positive impact on cut flowers. In addition, when cut lilies were treated with nifedipine alone, the vase life and HFO was significant suppressed. These results indicate that NO-delayed senescence might relative to Ca^2+^ and CaM, at least partly.

**Table 3:** Effects of Ca^2+^ chelators, Ca^2+^ channel inhibitors, and CaM antagonists on SNAP-regulated vase life, maximum flower diameter, and hours until full flower opening of cut OT hybrids lily “Manissa.”

Treatments	Vase life (d)	Maximum flower diameter (mm)	Hours until full flower opening (HFO, h)
Distilled water (control)	9.9 ± 0.6 b	185.64 ± 3.35 b	149.7 ± 11.1 b
SNAP	12.5 ± 0.1 a	221.33 ± 7.70 a	187.8 ± 6.5 a
SNAP + EGTA	10.3 ± 0.5 b	184.37 ± 8.32 b	157.7 ± 8.9 b
SNAP + BAPTA/AM	10.4 ± 0.4 b	190.05 ± 6.79 b	147.3 ± 4.2 b
SNAP + LaCl_3_	10.5 ± 0.3 b	201.77 ± 11.20 b	158.6 ± 10.2 b
SNAP + nifedipine	10.1 ± 0.3 b	197.46 ± 5.27 b	140.1 ± 3.6 b
SNAP + W-7	9.7 ± 0.3 b	203.69 ± 6.66 b	146.4 ± 9.7 b
SNAP + TFP	9.5 ± 0.4 b	180.73 ± 2.28 b	151.2 ± 6.2 b
Nifedipine	8.7 ± 0.1 c	183.20 ± 2.61 b	136.8 ± 3.6 c

*The values (mean ± SE) are the average of three independent experiments (n = 15 flowering stems). Values not sharing the same letters in the same list are statistically significant by Duncan’s multiple range test at 5% level.*

### Effects of Ca^2+^ Chelators, Ca^2+^ Channel Inhibitors, and CaM Antagonists on SNAP-Induced Calcium Level

**FIGURE 2:**
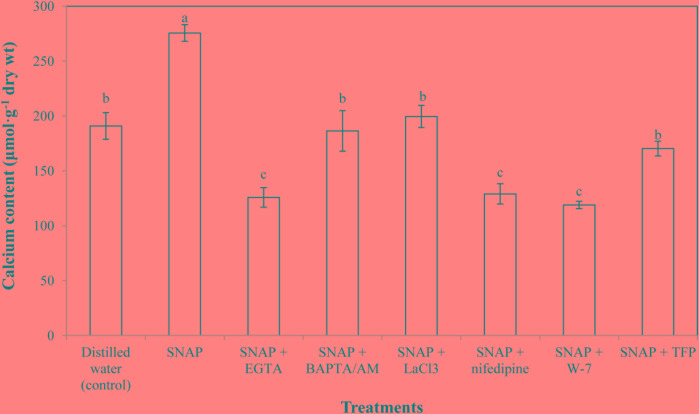
Effects of SNAP, Ca^2+^ channel inhibitors, Ca^2+^ chelators, and CaM antagonists on calcium content. SNAP, EGTA, BAPTA/AM, LaCl_3_, nifedipine, W-7, and TFP were used at 100 120, 30, 500, 150, 80, and 100 μM, respectively. Error bars represent standard error and each data in figure represents the mean ± SE of three experiments. Bars not sharing the same letters were significantly different by Duncan’s multiple range test (*P* < 0.05).

To investigate the possible roles of Ca^2+^ concerned with NO-induced effect during the senescence process, the Ca^2+^ content in cut lily flowers of treatments with SNAP or SNAP in combination with EGTA, BAPTA/AM, LaCl_3_, nifedipine, W-7, and TFP were tested. **Figure 2** shows that when SNAP applied alone, an obviously increase in Ca^2+^ level was detected, which was 144.45% of the control. When BAPTA/AM, LaCl_3_ or TFP was added to the SNAP-treated cut flowers, the effects of NO were weakened. However, SNAP treatment with EGTA, nifedipine or W-7 resulted in a significant reduction in Ca^2+^content. Placing CaM antagonists W-7 into SNAP solution maximally reversed the promotion induced by SNAP, which were 56.86% lower than that of the treatment with SNAP alone.

### Effects of Ca^2+^ Chelators, Ca^2+^ Channel Inhibitors, and CaM Antagonists on SNAP-Induced Calmodulin Level

**FIGURE 3:**
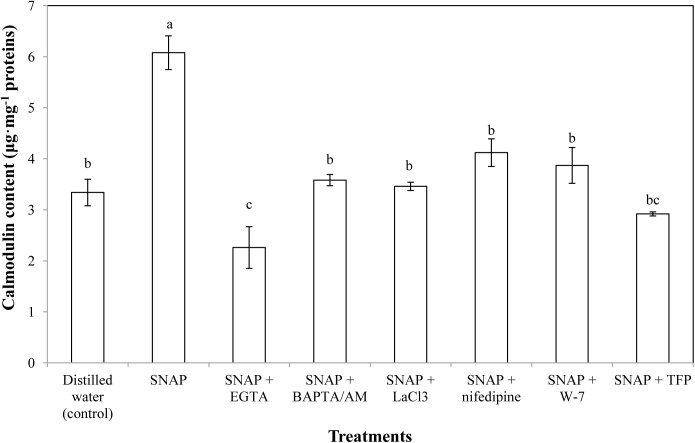
Effects of SNAP, Ca^2+^ channel inhibitors, Ca^2+^ chelators, and CaM antagonists on calmodulin content. NAP, EGTA, BAPTA/AM, LaCl_3_, nifedipine, W-7, and TFP were used at 100 120, 30, 500, 150, 80, and 100 μM, respectively. Error bars represent standard error and each data in figure represents the mean ± SE of three experiments. Bars not sharing the same letters were significantly different by Duncan’s multiple range test (*P* < 0.05).

As shown in **Figure 3**, the effects of different vase solutions (SNAP was applied alone or together with Ca^2+^ chelators, Ca^2+^ channel inhibitors or CaM antagonists) on CaM levels were determined. SNAP treatment gave the maximum CaM content, which was almost twice that of the control. The SNAP-induced increase on CaM content was inhibited by BAPTA/AM, LaCl_3_, nifedipine, W-7, or TFP, thereby resulted in similar contents to the control. Moreover, the vase solution including both SNAP and Ca^2+^ chelator EGTA caused a sharp decrease in endogenous CaM content, which was obviously lower than that of the control. These results indicate that SNAP promoted the CaM content of cut lily flowers, while Ca^2+^ chelators, Ca^2+^ channel inhibitors or CaM antagonists inhibited the effects.

### Effects of Ca^2+^ Chelators, Ca^2+^ Channel Inhibitors, and CaM Antagonists on SNAP-Induced Ca^2+^-ATPase Activity

**FIGURE 4:**
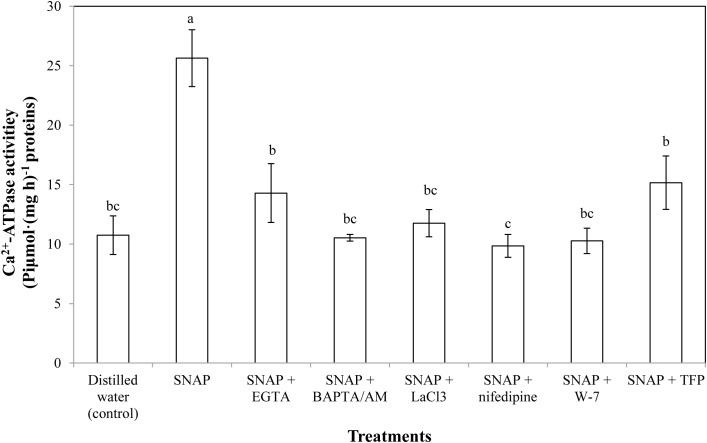
Effects of SNAP, Ca^2+^ channel inhibitors, Ca^2+^ chelators, and CaM antagonists on Ca^2+^-ATPase activity. NAP, EGTA, BAPTA/AM, LaCl_3_, nifedipine, W-7, and TFP were used at 100 120, 30, 500, 150, 80, and 100 μM, respectively. Error bars represent standard error and each data in figure represents the mean ± SE of three experiments. Bars not sharing the same letters were significantly different by Duncan’s multiple range test (*P* < 0.05).

There was a significant increase in the Ca^2+^-ATPase activity twice more than the control when SNAP applied alone (**Figure 4**). Placing EGTA, LaCl_3_, or TFP into SNAP solution partly reversed the promotion of SNAP. SNAP treatment with BAPTA/AM, nifedipine, or W-7 also caused a decrease in Ca^2+^-ATPase activity. The minimum Ca^2+^-ATPase activity were obtained by the SNAP plus Ca^2+^ channel inhibitor nifedipine, suggesting that nifedipine maximally prevented the NO-induced positive effects in the senescence process of cut lilies.

### Effects of Ca^2+^ Chelators, Ca^2+^ Channel Inhibitors, and CaM Antagonists on SNAP-Induced the Expression of Ca^2+^ Signaling-Related Genes

**FIGURE 5:**
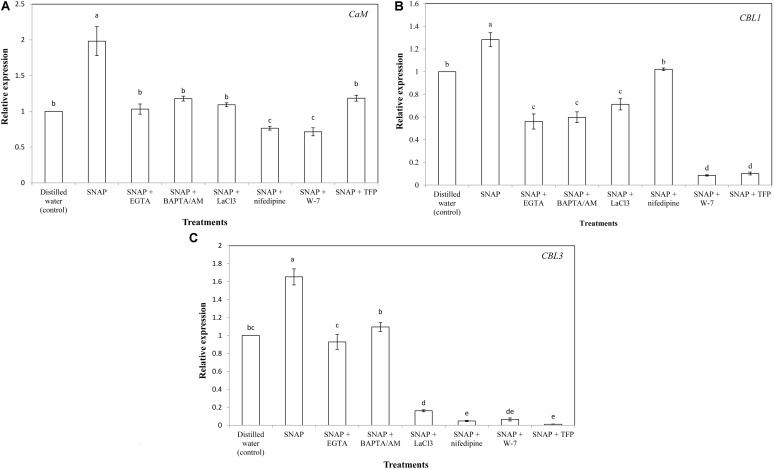
Effects of SNAP, Ca^2+^ channel inhibitors, Ca^2+^ chelators, and CaM antagonists on the expression levels of **(A)** *CaM*, **(B)** *CBL1*, and **(C)** *CBL3*. NAP, EGTA, BAPTA/AM, LaCl_3_, nifedipine, W-7, and TFP were used at 100 120, 30, 500, 150, 80, and 100 μM, respectively. Error bars represent standard error and each data in figure represents the mean ± SE of three experiments. Bars not sharing the same letters were significantly different by Duncan’s multiple range test (*P* < 0.05).

The gene expression of *CaM* was significantly increased by SNAP, resulted in a maximum value which was almost double the control; while the co-treatment with SNAP and EGTA, BAPTA/AM, LaCL_3_, nifedipine,W-7, or TFP obviously reduced the positive effects of SNAP (**Figure 5A**). There was no difference in CaM expression between the control and SNAP plus EGTA, BAPTA/AM, LaCL_3_, or TFP. However, SNAP treatment in combination with nifedipine or W-7 caused a significant decrease compared to the control. Placing SNAP into the vase solution also upregulated *CBL1* expression significantly, whereas there was a great inhibition when SNAP applied together with EGTA, BAPTA/AM, LaCL_3_, nifedipine, W-7, or TFP (**Figure 5B**). Especially when EGTA, BAPTA/AM, LaCL_3_, W-7, or TFP was applied to SNAP-treated cut lily flowers, the expression of *CBL1* was lower than that of the control. Similarly, EGTA, BAPTA/AM, LaCL_3_, nifedipine, W-7, or TFP obviously reduced the SNAP-induced increase on *CBL3* expression (**Figure 5C**).

## Discussion

Our results show that exogenous NO and Ca^2+^ at proper dose could help against cut lily flowers senescence, while high concentration of NO and Ca^2+^ were invalid. This conforms to our previous study, which demonstrated that the effects of NO on vase life and maximum flower diameter of cut roses were dose-dependent (Liao et al., 2013). In addition, the effects of NO on dormancy release in bulbs of oriental lily also depend on NO concentration (Niu et al., 2015). Thus, the dual role of NO as an effective antioxidant or potent oxidant is dependent on NO concentrations (Beligni and Lamattina, 1999). Exogenous NO at a relatively low dose could increase vase life of various kinds of cut flowers, such as roses (Liao et al., 2013), tuberose (Beni et al., 2013), gladiolus (Dwivedi et al., 2016), gerberas (Shabanian et al., 2018), sunflowers, lisianthus (Nazirimoghaddam et al., 2014), lilies, snapdragons, delphiniums, tulips, irises, chrysanthemums (Badiyan et al., 2004), pineapple lilies (Salachna and Byczyńska, 2017), and carnations (Naing et al., 2017). It also appears that Ca^2+^ plays a critical role during postharvest freshness. Exogenous Ca^2+^ is effective for quality preservation and disease resistance of apples (Zhao and Wang, 2015). Pre-harvest treatment with nano-calcium showed promoting effect on pepper fruits quality after plucking (Amini et al., 2016). The author also showed that Ca^2+^ improved total soluble solids, ascorbic acid, weight loss, electrolyte leakage, lipid peroxidation, and protein content. We used two controls (treatment with distilled water or KCl) to eliminate the influence of chloride ion and further identify the promoting effects of Ca^2+^ on postharvest life of cut lily flowers. The results suggests that Ca^2+^ enhanced vase life because KCl had no significant effects on postharvest freshness in cut lily flowers compared with the control, while CaCl_2_ significantly increased vase life, flower diameter and HFO. Our results also supported by the observation of authors who indicated that Ca^2+^ could significantly increase vase life of cut gerbera flowers by delaying stem bending (Perik et al., 2014). However, the optimum concentration of CaCl_2_ for longer vase life in the study of Perik et al. (2014) and our experiment are not the same. The most likely explanation for this observation is that different plant species show different sensitivities to Ca^2+^. Additionally, dissimilar concentration grads used in the two experiments may be another explanation.

The possibility that NO and Ca^2+^, as plant signaling regulators, may work together in response to abiotic stresses has been reported (Silveira et al., 2017). In the present study, when Ca^2+^ chelators, Ca^2+^ channel inhibitors, and CaM antagonists were applied, the promoting effects of NO on vase life was blocked. Meanwhile, the Ca^2+^ channel inhibitor nifedipine itself played negative roles in fresh-keeping by inhibiting endogenous Ca^2+^. According to our previous study, the Ca^2+^ chelators (EGTA, BAPTA/AM) and CaM antagonists (W-7, TFP) applied alone might inhibit adventitious rooting of marigold, while NO or Ca^2+^ promoted this process (Liao et al., 2012b). However, van Doorn et al. (2013) found that neither CaM antagonists W-7 nor TFP had an effect on visible senescence of cut iris flowers. Thus, these chemicals might have different roles in different physiological processes or in different plant species. There is, therefore, a definite need for studying the roles of these inhibitors or antagonists in plant growth and development. It should be noted that we did not investigate the effects of Ca^2+^ chelators and CaM antagonists on senescence alone in this study, and this should be carefully treated. Our results further show that Ca^2+^/CaM might be involved in the NO-regulated postharvest freshness process of cut lily flowers. The earlier reports about other plants, including cucumber (Niu et al., 2017), fava bean (Zhao et al., 2013), grape (Vandelle et al., 2006), tobacco (Lamotte et al., 2006), *Arabidopsis* (Abdul-Awal et al., 2016), tall fescue (Xu et al., 2016), tomato (Siddiqui et al., 2017), wheat (Tian et al., 2015), rice (Chen and Kao, 2012), and marigold (Liao et al., 2012b), are in conformity with our results, suggesting there are intricate interplays between NO and Ca^2+^.

**FIGURE 6:**
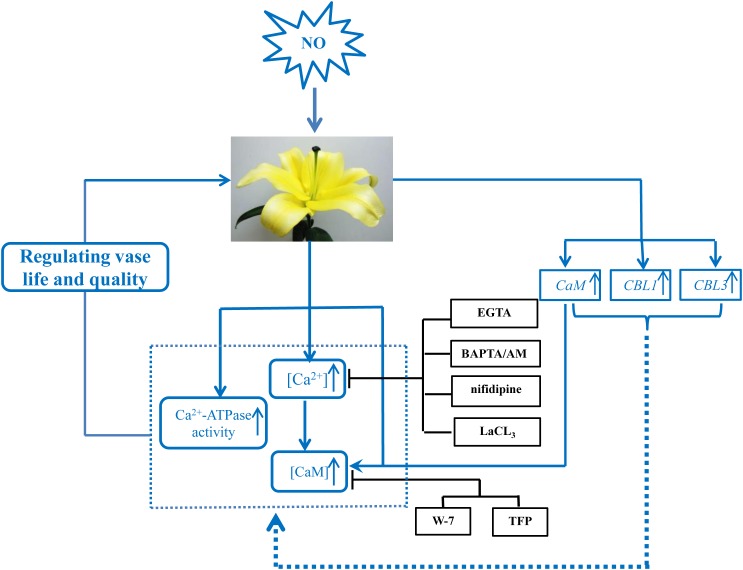
Schematic model of the signaling networks involving NO and Ca^2+^/CaM during postharvest freshness of cut lily flowers. NO induces the upregulation of Ca^2+^ signaling-regulated genes as well as the content of Ca^2+^/CaM and Ca^2+^-ATPase activity, as a consequence contributes to the increase of vase life. ↑, increase; ⊢, inhibition. All the inhibitors assayed in this study are black boxed.

Cytosolic free Ca^2+^ concentration ([Ca^2+^]_cyt_) variations have been reported in response to a large set of stimuli (Liao et al., 2012b). NO plays a role in elevating free [Ca^2+^]_cyt_ by the mobilization of intracellular pools of Ca^2+^, which appears to act through cyclic guanosine monophosphate (cGMP) and cyclic adenosine diphosphoribose (cADPR; Tian et al., 2015). In the present study, about 144.45% of increase in the accumulation of Ca^2+^ was observed in cut lilies treated with NO, while Ca^2+^ chelators, Ca^2+^ channel inhibitors, and CaM antagonists significantly reduced Ca^2+^ content in NO-treated flowers. The results indicate that Ca^2+^ was involved in the NO-related postharvest freshness, and the positive effect of NO might depend on increased Ca^2+^ content (**Figure 6**), at least partly. Recent studies from our laboratory have shown that intracellular Ca^2+^ amount was increased by NO in cucumber hypocotyls during the development of adventitious roots under osmotic stress (Niu et al., 2017). These results are in full agreement with the findings of Siddiqui et al. (2017), who found that exogenous NO and Ca^2+^ applied individually as well as in combination significantly enhanced of Ca^2+^ content under both heat stress and non-stress conditions. Interestingly, exogenous NO also could promote the selectivity of uptake and transport of Ca^2+^, the authors also found that Ca^2+^ could counteract the toxic effect of exogenous NO on root. Therefore, combined application of NO and Ca^2+^ could compensate for each other’s disadvantages to obtain a synergic effect in alleviating the adverse effects of salt stress (Tian et al., 2015).

To further investigate the relationships of NO and Ca^2+^, the CaM level was tested during senescence process of cut lily flowers. When NO applied exogenously, CaM content was almost double of the control, indicating that CaM was involved in the NO-related freshness preservation (**Figure 6**). This result was further confirmed by the use of Ca^2+^ chelators, Ca^2+^ channel inhibitors, and CaM antagonists, which resulted in a significant decrease in CaM content. This result is in conformity with our previous study which shows that the NO-induced CaM level was inhabited by Ca^2+^ chelators BAPTA or CaM antagonist W-7 (Liao et al., 2012b). Therefore, Ca^2+^/CaM may function as downstream signaling molecules of NO in the postharvest freshness of cut lily flowers. The evidence has shown that Ca^2+^/CaM might be both upstream and downstream of NO signaling cascades in both animals and plants (Weiner et al., 1994; Ali et al., 2007). Thus, different targets and/or circuitry for NO signaling must exist. Moreover, an active Ca^2+^ transport system, such as the Ca^2+^ pump (Ca^2+^-ATPase) on cellular membranes, is critical for maintaining Ca^2+^ homeostasis (Jian et al., 1999). Plant Ca^2+^-ATPase was also involved in biological process, such as hormonal regulation, pathogens response, and mineral nutrition/toxicity (Bonza and De Michelis, 2011). In the present study, Ca^2+^-ATPase activity was significantly increased when NO was applied, suggesting that Ca^2+^-ATPase is beneficial for NO-induced freshness preservation of cut flowers (**Figure 6**). A previous study shows that *CaM3* expression was induced by heat and CaCl_2_ and correlated positively with thermotolerance of lily (Cao et al., 2013). Our results showed exogenous NO increased the expression of *CaM* during cut lily postharvest freshness process, while Ca^2+^ chelators (EGTA, BAPTA/AM), Ca^2+^ channel inhibitors (LaCl_3_, nifedipine) and CaM antagonists (W-7, TFP) inhibited the NO-induced *CaM* gene expression. Interestingly, neither membrane-impermeable Ca^2+^ chelators EGTA nor membrane-permeable Ca^2+^ chelators BAPTA/AM inhibited *CBL3* expression as much as *CBL1* expression. Probably because CBL1 and CBL3 have different sensitivity to these two Ca^2+^ chelators and this possibility should be further investigated. Similarly, the *CBL1* and *CBL3* gene expression was also upregulated by NO, and the NO-enhanced gene expression was suppressed by these chelators, antagonists, or inhibitors (**Figure 6**). Notably, CaM antagonists W-7 and TFP also largely inhibited *CBL1* and *CBL3* expression. These results further verified the study of Anil and Rao (2000), who reported that the compounds TFP and W-7 were not specific for inhibiting CDPKs. TFP and W-7 also been reported to affect the activity of other Ca^2+^-binding proteins such as CaM and CBLs (Anil and Rao, 2000). Thus, these results suggested that NO might help cut lily flowers against senescence by inducing the Ca^2+^ signaling-regulated gene expression of *CaM*, *CBL1*, and *CBL3*.

## Conclusion

Altogether, the results of our study have shown that exogenous application of NO and Ca^2+^ could prolong vase life of cut flowers. NO might promote postharvest freshness by increasing Ca^2+^/CaM content, and regulating gene expression of Ca^2+^ signaling-regulated proteins. Our data also revealed that Ca^2+^/CaM might act downstream molecules of NO signaling pathway. However, the interaction between NO and Ca^2+^/CaM seems to be very complex. Therefore, more efforts are needed to demonstrate the detailed mechanism during senescence processes of cut flowers.

## Author Contributions

WL designed the experiment. JZ, HF, JH, and DH preformed the experiment. JZ, HF, JH, and BW performed the data analysis. JZ wrote the manuscript. WL revised the manuscript. All the authors read and approved the final manuscript.

## Conflict of Interest Statement

The authors declare that the research was conducted in the absence of any commercial or financial relationships that could be construed as a potential conflict of interest.
