# Supplementary material for: Involvement of Calcium and Calmodulin in Nitric Oxide-Regulated Senescence of Cut Lily Flowers
Source: Front Plant Sci. 2018 Sep 3;9:1284. doi: 10.3389/fpls.2018.01284 (PMC6129963; doi:10.3389/fpls.2018.01284)
Supplement: Supplementary file 1 [file Table_1.DOCX]

Table S1. Real-time PCR primers of calcium signaling related genes.

| Gene | Forward PCR (5′-3′) | Reverse PCR (5′-3′) |
| --- | --- | --- |
| *LlCaM* | GTAGATGCTGACGGCAATGG | AGCCATTCTGGTCCTTGTCA |
| *LlCBL 1* | GACCCGGTGCTTCTTTCTTC | CCAAGGAACTGCTGATGCTC |
| *LlCBL3* | ATTTGCTGACCGGGTGTTTG | GGCGCGTTAGGATGAAAGAC |
